# Synthesis, characterization, and antioxidant activity of Zn^2+^ and Cu^2+^ coordinated polyhydroxychalcone complexes

**DOI:** 10.1007/s00706-016-1822-7

**Published:** 2016-08-29

**Authors:** Chiara Sulpizio, Simon T. R. Müller, Qi Zhang, Lothar Brecker, Annette Rompel

**Affiliations:** 1 Institut für Biophysikalische Chemie, Fakultät für Chemie, Universität Wien, Althanstraße 14, 1090 Vienna, Austria; 2 Institut für Organische Chemie, Fakultät für Chemie, Universität Wien, Währinger Straße 38, 1090 Vienna, Austria

**Keywords:** Bioinorganic chemistry, Carbonyl compounds, Metal complex, NMR spectroscopy, Structure activity relationship

## Abstract

**Abstract:**

Four new metal complexes [Cu(ISO)_2_], [Cu(BUT)_2_] and [Zn(ISO)_2_], [Zn(BUT)_2_] of the polyhydroxychalcones (isoliquiritigenin and butein) are synthesized, structurally characterized and their antioxidant activity is investigated. The formation of the complexes [Cu(ISO)_2_] and [Zn(ISO)_2_] is followed by Job’s plot using NMR titration. The resulting compounds are characterized by mass spectrometry, IR spectroscopy, and elemental analysis. Studies on the radical scavenging activity are performed using DPPH as substrate. The results showed that the antioxidant activities of isoliquiritigenin and butein are enhanced after binding to copper or zinc.

**Graphical abstract:**

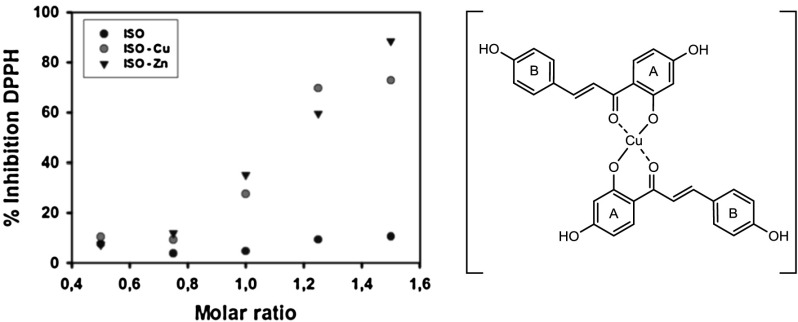

**Electronic supplementary material:**

The online version of this article (doi:10.1007/s00706-016-1822-7) contains supplementary material, which is available to authorized users.

## Introduction

**Fig. 1:**
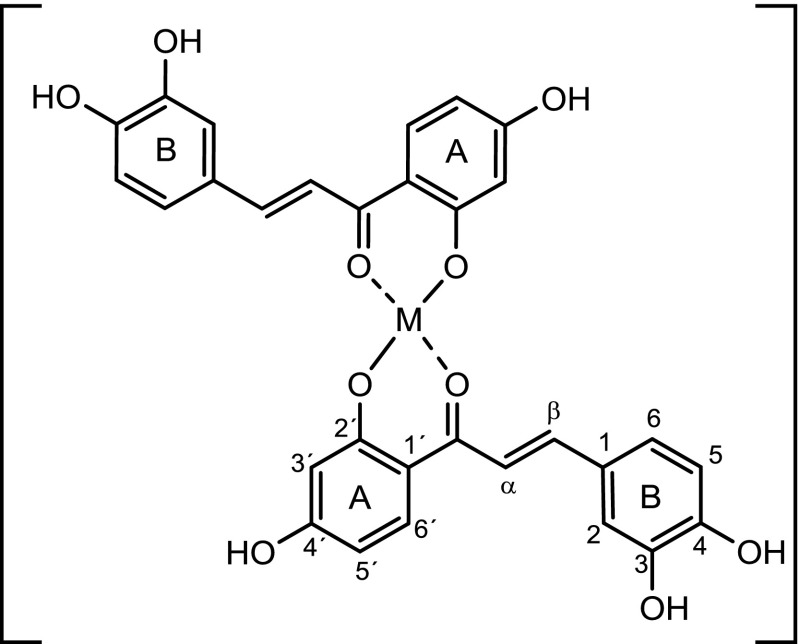
Proposed structure of a general M(II)–chalcone complex

Oxidation processes are essential for life, nevertheless they can have a toxic potential as they generate free radicals which can cause degradation in food, chemicals, and in biological systems [1–4]. Hence, antioxidants play a central role in human health as they scavenge potentially harmful free radicals such as reactive oxygen species (ROS). Chalcones, being a subgroup of the flavonoid family, have received considerable attention as radical scavengers and show an impressive range of biological activities such as antibacterial [5, 6], antiviral [7–9], and anti-inflammatory properties [10, 11]. Polyhydroxychalcones can be considered the most active chalcones due to their ability to form stabilized phenoxy radicals [11–14]. Hwang et al. [15] have shown that the position of the hydroxyl groups on arene ring B (Fig. 1) has a major impact on radical scavenging properties of polyhydroxychalcones. While *meta*-substitution on ring B has no beneficial effect, *ortho*- and *para*-substitutions increase the antioxidant activity of chalcones significantly, presumably due to the ability to form quinones as oxidation products.

Recently, it has been shown that the antioxidant activity of polyphenols, structurally similar to polyhydroxychalcones, can be significantly enhanced by metal ion complexation [16–19]. In contrast to the interest that metal complexes of polyphenolic substances have received, little is known about the synthesis and activity of polyhydroxychalcone metal complexes, and how metal complexation would impact the antioxidant activity of these powerful secondary plant metabolites. Although in literature three metal complexes of 2'-hydroxychalcones with Zn, Cd, and Hg are reported [20], no complexes of polyhydroxychalcone have been synthesized, isolated, and characterized until now. Thus in particular the possible structure of metal ion coordination needs to be investigated. There are several options how metals can coordinate polyhydroxychalcones (see Fig. 1) [21, 22]. Both, the carbonyl moiety and the 2′-hydroxyl group of the 2′-hydroxychalcones have been suggested to coordinate a metal(II) ion [23]. Different complex formation between polyhydroxychalcones and metal ions show various affects on the electronic distribution in the molecule, resulting in possible changes of biological properties such as radical scavenging ability.

**Figure:**
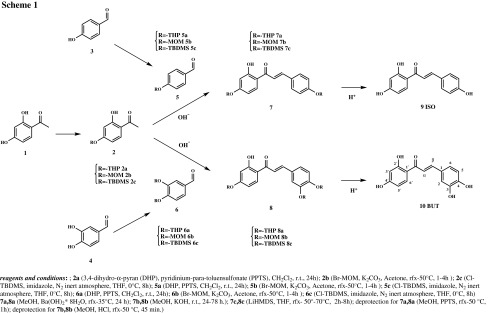

Copper(II) and zinc(II) are our metals of interest, since they are endogenous metals and they have shown promising features as metallodrug candidates [24, 25]. In fact Cu(II) and Zn(II) complexes of the flavonoids quercetin and rutin have been shown to greatly increase the antioxidant activity of this class of compounds [26, 27]. In many complexes Zn(II) prefers a tetrahedral coordination sphere [28] while the Cu(II) cation prefers a square planar coordination. Based on the polyhydroxychalcones scaffold other coordination geometries such as distorted octahedral geometry have been reported for copper [29]. In this communication we disclose synthesis, characterization as well as studies on antioxidant activities of four polyhydroxychalcone metal complexes.

## Results and discussion

### Synthesis of polyhydroxychalcone and their metal complexes

The synthesis of the polyhydroxychalcones was performed via a well-documented three-step procedure in basic condition according to [30] (see Scheme 1). First compound **1** was protected with one of the three protecting groups, methoxymethyl (MOM), tetrahydropyranyl (THP), and *tert*-butyldimethylsilyl-ether (*t*-BDMS) to get **2**. Protection proceeded in all cases in moderate to good yields. Subsequently, a base-catalyzed aldol condensation reaction (Claisen–Schmidt condensation) with a protected benzaldehyde **5** or **6** was performed to obtain the protected chalcones **7** and **8**. Finally, deprotection was performed to give the desired polyhydroxychalcones isoliquiritigenin (**9**, ISO) and butein (**10**, BUT).

In order to optimize the synthesis of the polyhydroxychalcones, different protecting groups have been applied in the course of the butein synthesis (Scheme 1). MOM as a protection group presents several disadvantages as it is highly carcinogenic [31], and the purification of the protected intermediate with liquid column chromatography is challenging. Nevertheless, the protection reaction of the hydroxyl group with MOM leads to a yield of 67 % for benzaldehyde and 64 % for acetophenone. Moreover, the removal of the MOM group requires drastic acidic conditions [32], which often lead to polymerization and oxidation of the desired product. *t*-BDMS worked better during the protection step providing similar and higher yields (64 % for the benzaldehyde and 83 % for the acetophenone). The coupling was performed using LiHMDS [33], but difficulties with the purification made isolation of the pure product problematic. Although THP is a little less efficient in protecting benzaldehyde (60 % yield) it protects the acetophenone most efficiently (89 %), and has been advantageous in all other aspects as it is easy to insert and remove. Furthermore, it is stable under the alkaline coupling conditions [30]. As a consequence THP was chosen as the protection group for the synthesis of both polyhydroxychalcones ISO and BUT.

Independently of the used protection group, all the condensation reactions worked with moderate yields of 45 and 52 %, respectively (see Table 1). For some substrates MOM-ether worked better during the deprotection step than the THP-ether [34]. Nevertheless, this strongly depended on the substrate as for some THP-ether provided better results. The overall yields for the three steps were 15 % for the MOM protecting group and 11 % for the THP protecting group (see Table 1). These overall yields are in accordance with the work published by Lee et al. [35], who obtained the final hydroxychalcone in an overall yield of 10 % when they protected the 4-hydroxy group with methoxymethyl prior to the Claisen–Schmidt condensation. It should be noted that the use of a Sephadex LH-20 for liquid column chromatography to purify the final polyhydroxychalcone increased the yield significantly compared to a silica gel-based liquid chromatography.

**Table 1:** Comparison of protecting groups for synthesis of butein

Protecting group, yield/%	Protection benzaldehyde, yield/%	Protection acetophenone, yield/%	Coupling reaction, yield/%	Deprotection, yield/%	Overall, yield/%
MOM	67	64	45	55	15
THP	60	89	52	25	11
*t*-BDMS	64	83	<15	n.d.	n.d.

*n.d.* not determined

The two complexes [Cu(ISO)_2_] and [Cu(BUT)_2_] were prepared by mixing copper(II) acetate with (*E*)-1-(2,4-dihydroxyphenyl)-3-(4-hydroxyphenyl)-2-propen-1-one (**9**, ISO) and (*E*)-1-(2,4-dihydroxyphenyl)-3-(3,4-dihydroxyphenyl)-2-propen-1-one (**10**, BUT), respectively, in a 1:2 (M, M = metal: L, L = ligand) molar ratio. The reactions were carried out in methanol as solvent.

The complexes [Zn(ISO)_2_] and [Zn(BUT)_2_] were prepared by mixing zinc(II) chloride with (*E*)-1-(2,4-dihydroxyphenyl)-3-(4-hydroxyphenyl)-prop-2-en-1-one (**9**, ISO) and (*E*)-1-(2,4-dihydroxyphenyl)-3-(3,4-dihydroxyphenyl)prop-2-en-1-one (**10**, BUT) in a 1:2 (M:L) molar ratio. The reactions were carried out in acetonitrile. Four new Cu(II) and Zn(II) complexes of polyhydroxychalcones termed [Cu(ISO)_2_], [Cu(BUT)_2_], [Zn(ISO)_2_], and [Zn(BUT)_2_] have been obtained with yields of about 50 %.

### Analysis of the polyhydroxychalcone metal complexes

The polyhydroxychalcones remain structurally unchanged in the metal complexes, as exemplarily shown by NMR data of compound **9**, ISO. In particular, the metal complexes exist in the *E*-form, which was confirmed by the characteristic coupling constant between the α and β protons of the double bond [see ^1^H NMR of (**9**, ISO), *J* = 15.4 Hz, Fig. 3, ratio 10:0]. Despite several attempts we were not able to obtain single crystals of [Cu(ISO)_2_], [Zn(ISO)_2_], [Cu(BUT)_2_], and [Zn(BUT)_2_].

### Stoichiometry of the metal complexes using Job’s plot

**Fig. 2:**
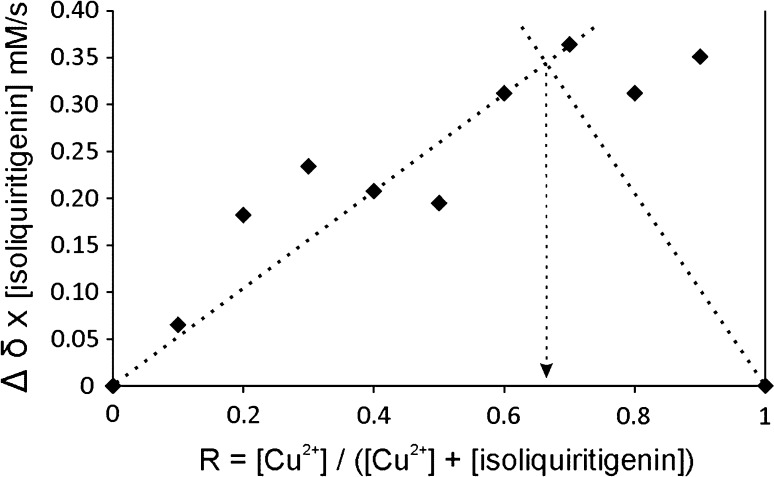
Job’s plot determined from the chemical shift changes of H-5′ of isoliquiritigenin (**9**, ISO) with Cu(OAc)_2_ in methanol-*d*_*4*_. Summed concentration of isoliquiritigenin (**9**, ISO) and Cu(OAc)_2_ is 13 mM

Studies to explore the stoichiometry between the polyhydroxychalcones and Zn^2+^ and Cu^2+^ were performed using Job’s plot on the basis of ^1^H NMR measurements [36]. The product concentration of the metal ion and the shift difference of some indicative protons was plotted against the molar fraction (see Fig. 2). Chemical shift changes of H-5′ of **9**, ISO (6.42 ppm) were measured as a function of molar fraction. The rather large deviation in the region of the low isoliquiritigenin (**9**, ISO) concentration is caused by quite moderate *S*/*N* ratio and in particular by reasonable line broadening. The latter is likely caused by a higher amount of compound **9** being involved in complex formation with Cu(II) bearing unpaired electrons. The maximum at a mole fraction of ca. 0.66 is, however, even detectable in this case and indicates a 1:2 stoichiometry of the investigated complex (Fig. 2). Similar measurements with Zn(II) provide comparable results with an even better *S*/*N* ratio and less line broadening. Hence, isoliquiritigenin (**9**, ISO) coordinates to Zn^2+^ and Cu^2+^ in a ratio of 2:1 ISO:metal.

**Fig. 3:**
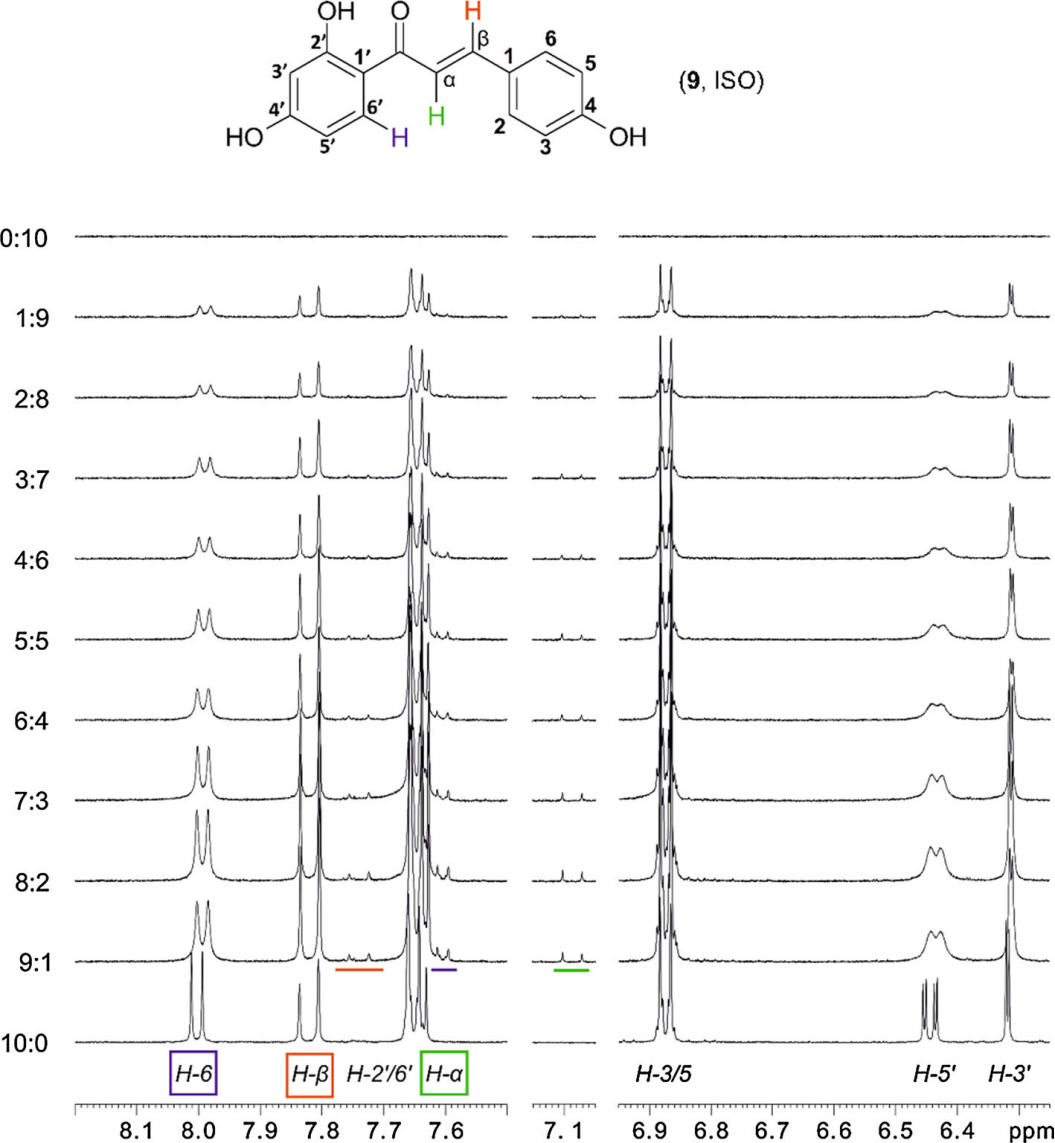
^1^H NMR titration of the Job’s plot of isoliquiritigenin (**9**, ISO) and Zn^2+^ with increasing amount of Zn^2+^ from the bottom to the top (methanol-*d*_*4*_). *Colored indications* highlight additional small signals attributed to H_6′_, H_β_, and H_α_ (color figure online)

The predominately shifted signals are attributed to the hydrogen of the double bond (H_α_, H_β_) and aromatic H (H_3′_, H_5′_, and H_6′_) on ring A (see Figs. 1 and 3), which confirm the keto function and the hydroxyl group at position C-2′ to be very likely involved in the coordination process. The NMR complexation studies give further insights on the side where the complexation occurs by showing a more distinct line broadening of signals from protons influenced by the metal ions (Fig. 3). These are in particular H_5′_ and H_6′_, as the hydroxyl group C-2′ ring A is involved in binding.

Addition of Zn(OAc)_2_ to the free ligand leads to the formation of some new small peaks which are shifted into a more shielded area in the ^1^H NMR (Fig. 3), likely indicating a side reaction induced by the complexation.

In all attempts to record a Job’s plot with butein (**10**, BUT) the resulting complexes were not completely soluble and a pale yellow precipitate was formed after certain time. Hence no ^1^H NMR spectra for the complex between butein and zinc as well as between butein and copper can be presented. Additionally ^13^C NMR studies have been carried out to investigate possible coordination modes but the analysis did not show any significant shift originating from metal complexation. These observations are in accordance with previous published data where the NMR spectra of the ligand in the presence of the metal do not show any differences from the pure ligand spectrum [37]. A possible explanation is that the butein–metal complex is removed from the solution due to precipitation.

### Mass spectrometry

Electrospray ionization mass spectrometry (ESI-MS) is performed on the Cu^2+^ and Zn^2+^ complexes of butein (**10**, BUT) and isoliquiritigenin (**9**, ISO), respectively. The ESI spectra show a typical isotope splitting matching the masses of ligand to metal complexes with a stoichiometry of 2:1, confirming their existence.

**Fig. 4:**
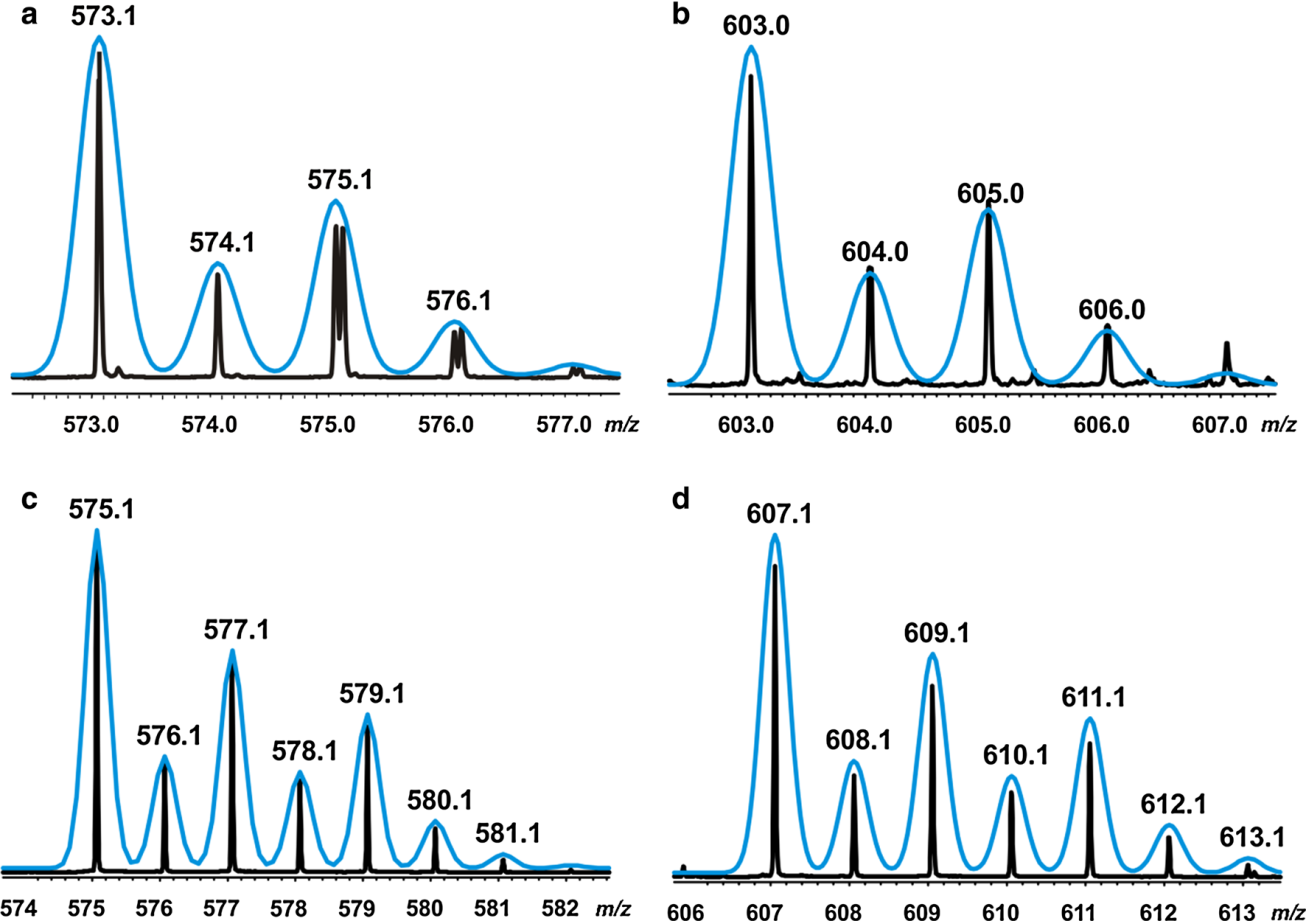
Electrospray ionization mass spectra of Cu^2+^/polyhydroxychalcones 1:2 system for [Cu(ISO)_2_] (**a**) in negative mode, [Cu(BUT)_2_] (**b**) in positive mode. The spectra **a** and **b** show the typical isotopic spectral pattern of the experimentally observed copper complexes Cu/L 1:2 in comparison with the corresponding calculated one (*blue*). The spectra **c** and **d** show the typical isotopic spectral pattern of the experimentally observed zinc complexes Zn/L 1:2 in comparison with the corresponding calculated one (*blue*) (color figure online)

For [Cu(ISO)_2_] these peaks can be found at *m/z* = 573.0612 for ^63^Cu and at 575.0604 for ^65^Cu (see Fig. 4). The peak corresponding to the free ligand was observed at *m/z* = 255.0664 in negative mode and also the adduct of two isoliquiritigenin molecules [(2 ISO-H^+^)] is present at *m/z* = 511.1391. In the case of butein the peaks of the copper adduct are present at 603.0362 for ^63^Cu and 605.0404 for ^65^Cu (see Fig. 4). These masses are attributed to the formula [Cu(C_15_H_10_O_5_)_2_]^2+^ indicating that the butein coordinates the copper ions in the oxidized form thereby explaining the loss of two Hs and we assumed that the catechol functionality on the B ring is oxidized to the *o*-chinone (catecholase activity, E.C. 1.10.3.1). Oxidation of flavonols may occur after copper complexation [38, 39]. The presence of the two peaks at *m/z* = 271.0599 (C_10_H_15_O_5_+H^+^) and 293.0422 (C_10_H_15_O_5_+Na^+^) results from the oxidized butein.

For [Zn(ISO)_2_] the peaks confirming a stoichiometry of two ligands per one zinc ion are found at *m/z* = 575.0676 (^63^Zn^2+^) and 577.0656 for ^65^Zn^2+^, while for [Zn(BUT)_2_] these peaks are observed at *m/z* = 607.0576 for ^63^Zn^2+^ and 609.0564 for ^65^Zn^2+^. The mass spectra did not show higher coordinated metal complexes of stoichiometry 3:1 or 4:1 (ligand to metal). There is no peak detectable, indicating a 1:1 stoichiometry of ligand to metal.

### IR spectroscopy

Infrared spectra of the Cu^2+^ and Zn^2+^ compounds were recorded and compared to the free ligands butein (**10**, BUT) and isoliquiritigenin (**9**, ISO). The intensity of the OH-vibration at 3300 cm^−1^ is reduced and broadened for both copper complexes, indicating that one or more hydroxyl groups could be interacting with the metal center [29]. Interestingly, the effect is more pronounced in the case of butein possessing the catechol type functionality on ring B. There is a shift of the carbonyl stretch from 1640 to 1610 cm^−1^ for both ligands in the presence of copper, meaning that the bond strength of the C=O bond is weakened. This is a strong indication for a participation of the carbonyl moiety in the complexation process [40]. Additionally, a new band is observed at 500 cm^−1^ in the spectra of the two metal complexes. In both cases these bands are attributed to the *ν* (Cu–O). This result is in accordance with the disclosure of McWhinnie [41], who has previously assigned the copper oxygen vibration at 500 cm^−1^ for related hydroxy-bridged copper compounds.

The IR spectra of the two polyhydroxychalcones with Zn^2+^ are quite complex as they in general exhibit a large number of bands of varying intensities. The IR spectra of isoliquiritigenin with Zn^2+^ do not show any significant shift compared to the free ligand, while the butein–Zn^2+^ complex shows a shift of the C=O stretching mode from 1630 to 1610 cm^−1^, indicating coordination of carbonyl oxygen to metal ion [42–44]. In combination with the results obtained from our ^1^H NMR titration studies, the IR data support the formation of the complex on the 2′-hydroxy/carbonyl side.

### Free radical scavenging ability of metal chalcone complexes

The antioxidant activity of the metal polyhydroxychalcones complexes has been evaluated applying a 1,1-diphenyl-2-picrylhydrazyl (DPPH) radical assay test, which is one of the most common methods used to determine radical scavenging ability. The DPPH test was chosen as it is a non-enzymatic method widely used to provide basic information on the capability of compounds in scavenging free radicals. It has already been proven to be a reliable method for flavonoids and other organic compounds. The test is performed in methanol because it is well known that DPPH is not soluble in water [45–47]. ESI, NMR, and IR spectroscopic investigations on the complexes point towards metal coordination at the keto enol moiety of the polyhydroxychalcones. Thus, the hydroxy groups are able to participate in the free radical scavenging activity. The activity of the metal complexes of isoliquiritigenin (**9**, ISO) with copper and zinc and of butein (**10**, BUT) with copper have been measured and compared with the one of the free ligands. Catechol has been used as a positive control and studies were performed on the system catechol-Cu^2+^ and catechol-Zn^2+^ as the free radical scavenging activity of phenols is not always enhanced upon metal binding [48]. Blanks of the sole metals Cu^2+^ and Zn^2+^ have also been carried out, too. The results are summarized in Table 2.

**Table 2:** The percentage of inhibition of the free DPPH radical in the presence of different Me-polyhydroxychalcone systems at 1.5 molar ratio (mol Me-polyhydroxychalcone/mol DPPH) for the studies with isoliquiritigenin (**9**, ISO) and the Me-isoliquiritigenin system and 0.75 molar ratio for the butein (**10**, BUT) and the Cu-butein system

Compound	DPPH radical scavenging activity/% inhibition
ISO	10
ISO-Cu^2+^	72
ISO-Zn^2+^	88
BUT	19
BUT-Zn^2+^	66
BUT-Cu^2+^	–
Catechol	94
Catechol-Zn^2+^	94
Cu^2+^	11
Zn^2+^	11

A solution of the Me-polyhydroxychalcone system at a stock concentration of 1.2 mM (0.8 mM of ligand and 0.4 mM of metal) has been used. Positive controls with Cu^2+^, Zn^2+^, catechol, and catechol-Zn^2+^ have been performed at 1.5 molar ratio

Cu^2+^ and Zn^2+^ ions coordinated to the keto enol functionality of isoliquiritigenin (**9**, ISO) alter the antioxidant activity of the chalcone. In fact at the final molar ratio of 1.5 (0.8 mM of ligand and 0.4 mM of metal) the metal complexes [Cu(ISO)_2_] and [Zn(ISO)_2_] show 72 and 88 % inhibition of the DPPH radical, whereas the sole ligand shows 10 % inhibition. A similar behavior is observed for butein (**10**, BUT) when complexating with Cu^2+^ (Table 2). The scavenging effect of the [Cu(ISO)_2_] was higher than that one of the sole ligand ISO. For [Cu(BUT)_2_] the free radical scavenging activity could only be observed up to a 0.75 molar ratio with 66 % inhibition in the presence of copper while the crude butein (**10**, BUT) shows only 19 % inhibition. In the preparation of the system butein/zinc a red precipitate was immediately formed, therefore it was not possible to perform the measurements. Pekal et al. [49] provided the antioxidant properties of a Cu^2+^/quercetin complex and Bratu et al. [26] on a Zn^2+^/rutin complex using the DPPH method. Both studies conclude that the radical scavenging activity of the metal flavonoid-complexes is higher than the one of the ligands alone. The results obtained support the mechanism suggested by Souza et al. [21]. The higher antioxidant activity of the complexes is due to the acquisition of additional superoxide dismutating centers, which causes an increase of the molecule’s ability to stabilize unpaired electrons and therefore to scavenge free radicals.

### Solubility and kinetic stability of [Cu(ISO)_2_], [Zn(ISO)_2_], [Cu(BUT)_2_], and [Zn(BUT)_2_]

The synthesized complexes [Cu(ISO)_2_], [Zn(ISO)_2_], [Cu(BUT)_2_], and [Zn(BUT)_2_] are insoluble in water only. Solubility is achieved in the presence of organic solvents. In fact the four complexes are soluble in methanol and dimethyl sulfoxide. 35 % of water is the maximum of water that is tolerated in the presence of dimethyl sulfoxide and methanol until a precipitate is formed.

Several methods for a spectrophotometric determination of the stability of metal complexes of flavonoids and related structures have been described in the literature. We chose the method described by Ferrari et al. [50] because the reproducibility and accuracy are satisfactory, and it has already been used to assess the kinetic stability of  a similar class of natural compounds such as curcumin derivatives. We tested the stability of the complexes under physiological conditions. Figures S1 and S2, reported in the Supplementary Material, show the decomposition kinetics profile of the four complexes and their linear fittings. Table S1 in the supporting information summarizes the hyperbolic function parameters (*a* and *b*) together with their reciprocal values and statistical parameters *R* ^2^. The reciprocal of *a* (1/*a*) is the percentage of residual compounds that remains intact under physiological conditions. The value (−1/*b*) is related to the degradation rate: the higher the value the faster the degradation process. [Zn(ISO)_2_] is characterized by the lowest values of 1/*a* and −1/*b*. In fact it decomposes up to ∼40 % in 16 h, and a similar value of ∼45 % is observed for [Cu(ISO)_2_]. [Cu(BUT)_2_] and [Zn(BUT)_2_] posses the highest values for 1/*a* which are 0.64 and 0.74, respectively. They are the most stable compounds, as after 8 h only 20 and 30 % degraded vs. 40 and 45 % of [Zn(ISO)_2_] and [Cu(ISO)_2_], respectively. This data points towards a relationship between the aromatic substituents and the kinetics profiles. In particular, the presence of an *ortho*/*para* substitution on the B ring seems to be fundamental in order to achieve stability. This is probably due to the possibility to form an intramolecular hydrogen bond, while the hydroxyl group on the A ring looks to be of minor importance. Therefore, the substitution pattern on the aromatic rings seems to be a key factor in slowing the degradation process of this class of metal complexes. At physiological pH, they have a kinetic stability comparable with other metal complexes of isoflavonoids [49].

## Conclusion

In this work, two polyhydroxychalcones isoliquiritigenin (ISO) and butein (BUT) were synthesized by Claisen condensation and characterized applying different spectroscopic and spectrometric techniques. The two chalcones have been reacted with Cu^2+^ and Zn^2+^ ions leading to the coordination compounds: [Cu(ISO)_2_], [Zn(ISO)_2_], [Cu(BUT)_2_], and [Zn(BUT)_2_].

The Job’s plot for the system isoliquiritigenin/metal(II) (metal = Cu^2+^, Zn^2+^) showed that isoliquiritigenin (**9**, ISO) forms a complex in solution with stoichiometry two to one, with the metal coordinating the keto function and the hydroxyl group at position C-2′. The IR and the mass spectra confirmed that both isoliquiritigenin and butein form stable complexes with copper and zinc. The metals coordinate through the carbonyl function and the hydroxyl function in *ortho*-position on ring A, again indicating that zinc and copper coordinate the polyhydroxychalcones with a ligand/metal stoichiometry of 2:1.

Finally, the antioxidant activity of the metal polyhydroxychalcones was determined with the DPPH test. The investigated complexes [Cu(ISO)_2_], [Zn(ISO)_2_], and [Zn (BUT)_2_] possess higher antioxidant activity than the free ligands isoliquiritigenin and butein.

## Experimental

Chemicals were purchased from Sigma Aldrich, they were of reagent grade and used without any further purification. All NMR spectra were recorded in the NMR Core Facility of the University of Vienna with a Bruker Avance III 500 MHz NMR spectrometer at 500.32 (^1^H), 125.81 (^13^C), in CDCl_3_ or methanol-*d*_4_, at ambient temperature. The splitting of proton resonances in the ^1^H NMR spectra are defined as s = singlet, d = doublet, dd = doublet of doublets, ddd = doublet of doublets of doublets, t = triplet, and m = multiplet. Numbering of carbon atoms and proton refers to Fig. 1 and Scheme 1. Elemental analyses were carried out in the Mikroanalytisches Laboratorium of the University of Vienna using an “EA 1108 CHNS-O” elemental analyzer by Carlo Erba Instruments. Electrospray ionization mass spectra were recorded in the Massenspektrometriezentrum of the University of Vienna with a Bruker Esquire 3000 with an orthogonal ESI source with MeOH/ACN as solvent, the molecular mass was determined in the positive as well as in the negative mode. The spectrophotometer Shimadzu UV 1800 has been used to recorder the UV–Vis spectra. The infrared spectra were recorded with an infrared spectrometer Bruker Tensor 27 FTIR equipped with a globar MIR light source, a KBr beam splitter, and a DLaTGS detector. Sample and background spectra were averaged from 100 scans at 4 cm^−1^ resolution. Undiluted sample powder was pressed on the diamond window of a Harrick MVP 2 diamond ATR accessory. Background spectra were obtained from the empty ATR unit. Data handling was performed with OPUS 5.5 software (Bruker Optik GmbH, 2005).

### Synthesis of the polyhydroxychalcones

(*E*)-1-(2,4-Dihydroxyphenyl)-3-(4-hydroxyphenyl)-2-propen-1-one and (*E*)-1-(2,4-dihydroxyphenyl)-3-(3,4-dihydroxyphenyl)-2-propen-1-one were synthesized performing a Claisen–Schmidt condensation between the benzaldehyde and acetophenone derivatives according to the previously published procedure [30]. The 3,4-dihydro-α-pyran is used to protect the hydroxyl group present on the aromatic ring before performing the aldol reaction in basic condition. The yields of each reaction are summarized in Table 1. The synthesized compounds were characterized by ^1^H NMR, mass spectrometry, and IR.

### 2-Hydroxy-4-(tetrahydro-2H-pyran-2-yloxy)acetophenone (**2a**, C_13_H_16_O_4_)

2,4-Hydroxyacetophenone (**1**, 6.60 mmol) and pyridinium *para*-toluenesulfonate (0.16 mmol) were dissolved in 30 cm^3^ of dichloromethane and the solution was stirred at room temperature. Subsequently, 3,4-dihydro-α-pyran (19.73 mmol) was added dropwise. The reaction was stirred for 24 h and the progress was monitored by TLC. Then the mixture was washed with water, dried over Na_2_SO_4_, concentrated under vacuum, and purified by flash column chromatography (hexane/ethyl acetate 9:2) to obtain pure **2a**. ^1^H NMR (500 MHz, CDCl_3_): *δ* = 12.62 (s, 1H, Ar–OH), 7.65 (d, *J* = 8.8 Hz, 1H, H-6), 6.63 (d, *J* = 2.5 Hz, 1H, H-3), 6.57 (dd, *J* = 8.8, 2.5 Hz, 1H, H-5), 5.49 (t, *J* = 3.1 Hz, 1H, H-7), 3.85 (ddd, *J* = 11.3, 9.9, 3.1 Hz, 1H, H-11a), 3.66–3.61 (m, 1H, H-11b), 2.58 (s, 3H, H-α), 2.04–1.95 (m, 1H, H-8a), 1.90–1.85 (m, 1H, H-8b), 1.75–1.65 (m, 2H, H-9a, H-9b), 1.65–1.59 (m, 1H, H-10a), 1.58–1.52 (m, 1H, H-10b) ppm; ^13^C NMR (125 MHz, CDCl_3_): *δ* = 202.6 (C=O), 164.8 (C-2), 163.1 (C-4), 132.3 (C-6), 114.5 (C-1), 108.5 (C-5), 103.4 (C-3), 96.1 (C-7), 63.0 (C-11), 30.6 (C-8), 30.0 (C-α), 25.4 (C-10), 19.7 (C-9) ppm; HRMS (ESI-MS): *m/z* = 259.0931 ([M+Na]^+^), 237.1452 ([M+H]^+^, calcd. for C_13_H_16_O_4_ 237.1121).

### 4-(Tetrahydro-2H-pyran-2-yloxy)benzaldehyde (**5a**, C_12_H_14_O_3_)

4-Hydroxybenzaldehyde (6.60 mmol) and pyridinium *para*-toluenesulfonate (0.16 mmol) were dissolved in 30 cm^3^ of dichloromethane and the solution was stirred at room temperature. Subsequently, 3,4-dihydro-α-pyran (19.73 mmol) was added dropwise. The reaction was stirred for 24 h and the progress was monitored by TLC. Then the mixture was washed with water, dried over Na_2_SO_4_, concentrated under vacuum, and purified by flash column chromatography (hexane/ethyl acetate 7:3) to obtain pure **5a**. ^1^H NMR (500 MHz, CDCl_3_): *δ* = 9.97 (s, 1H, C=O), 7.87–7.81 (dd, *J* = 8.9, 2.0 Hz, 2H, H-2, H-6), 7.20–7.13 (dd, *J* = 8.9, 2.0 Hz, 2H, H-3, H-5), 5.55 (t, *J* = 3.1 Hz, 1H, H-7), 3.93–3.82 (m, 1H, H-11a), 3.64 (m, 1H, H-11b), 2.08–1.96 (m, 1H, H-8a), 1.93–1.82 (m, 2H, H-8b, H-9a), 1.80–1.60 (m, 2H, H-9b, 10a), 1.58–1.50 (m, 1H, H-10b) ppm; ^13^C NMR (125 MHz, CDCl_3_): *δ* = 191.1 (C=O), 161.7 (C-4), 132.4 (C-2,6), 131.9 (C-1), 115.9 (C-3,5), 96.1 (C-7), 63.0 (C-11), 30.0 (C-8), 25.3 (C-10), 19.6 (C-9) ppm; HRMS (ESI-MS): *m/z* = 229.0829 ([M+Na]^+^, calcd. for C_12_H_14_O_3_Na 229.0835).

### 3,4-Bis(tetrahydro-2H-pyran-2-yloxy)benzaldehyde (**6a**, C_17_H_22_O_5_)

3,4-Dihydroxybenzaldehyde (6.60 mmol) and pyridinium *para*-toluenesulfonate (0.16 mmol) were dissolved in 30 cm^3^ of dichloromethane and the solution was stirred at room temperature. Subsequently, 3,4-dihydro-α-pyran (39.46 mmol) was added dropwise. The reaction was stirred for 24 h and the progress was monitored by TLC. Then the mixture was washed with water, dried over Na_2_SO_4_, concentrated under vacuum, and purified by flash column chromatography (hexane/ethyl acetate 9:2) to obtain **6a** as a mixture of stereoisomers. ^1^H NMR (500 MHz, CDCl_3_): *δ* = 9.87 (s, 1H, CHO), 7.50–7.49 (d, *J* = 8.0 Hz, 1H, H-6), 7.25 (s, 1H, H-2), 7.07–7.05 (d, *J* = 8.2 Hz, 1H, H-5), 5.55 (t, *J* = 2.7 Hz, 2H, H-7, H-7′), 3.93–3.82 (m, 2H, H-11a, H-11′a), 3.64 (m, 2H, H-11b, H-11b), 2.08–1.96 (m, 2H, H-8a, H-8a′), 1.93–1.82 (m, 4H, H-8b, H-8b′, H-9a, H-9a′), 1.80–1.60 (m, 4H, H-9b, H-9b′, H-10a, H-10a′), 1.58–1.50 (m, 2H, H-10b, 10b′) ppm; ^13^C NMR (125 MHz, CDCl_3_): *δ* = 190.6 (C=O), 153.1 (C-4), 146.9 (C-3), 129.8 (C-1), 123.9 (C-6), 115.8 (C-5), 115.2 (C-2), 98.4 (C-7), 98.9 (C-7′), 63.7 (C-11), 63.3 (C-11′), 30.1 (C-8), 30.1 (C-8′), 25.4 (C-10), 25.2 (C-10′), 19.4 (C-9), 18.8 (C-9′) ppm; HRMS (ESI-MS): *m/z* = 329.1356 ([M+Na]^+^), 307.1534 ([M+H]^+^, calcd. for C_17_H_22_O_5_ 307.1461).

### (*E*)-1-[2-Hydroxy-4-(tetrahydro-2H-pyran-2-yloxy)phenyl]-3-[4-(tetrahydro-2H-pyran-2-yloxy)phenyl]prop-2-en-1-one (**7a**, C_25_H_28_O_6_)

Compound **2a** (4.24 mmol) and 8.48 mmol of **5a** were dissolved in 10 cm^3^ of methanol at 35 °C under reflux. Subsequently, a solution of 20 cm^3^ of methanol containing barium hydroxide octahydrate (16.96 mmol) was added dropwise to the reaction mixture. The reaction mixture was stirred for 24 h and the progress was monitored by TLC. Then the mixture was concentrated under vacuum, quenched with HCl (0.1 M), and extracted with ethyl acetate. The organic layer was separated, dried over Na_2_SO_4_, and then concentrated under vacuum. The reaction mixture was purified by flash column chromatography (hexane/ethyl acetate 7:3) to obtain pure **7a**. ^1^H NMR (500 MHz, methanol-*d*_4_): *δ* = 8.07 (d, *J* = 8.8 Hz, 1H, H-6′), 7.85 (d, *J* = 15.4 Hz, 1H, H-β), 7.73 (d, *J* = 8.8 Hz, 2H, H-2, H-6), 7.70 (d, *J* = 15.4 Hz, 1H, H-α), 7.14 (d, *J* = 8.8 Hz, 2H, H-3, H5), 6.67 (dd, *J* = 8.8, 2.5 Hz, 1H, H-5′), 6.62 (d, *J* = 2.5 Hz, 1H, H-3′), 5.58 (t, *J* = 3.1 Hz, 1H, H-7), 5.53 (t, *J* = 3.1 Hz, 1H, H-7′), 3.92–3.80 (m, 2H, H-11a, H-11a′), 3.69–3.59 (m, 2H, H-11b, H-11b′), 2.07–1.96 (m, 2H, H-8a, H-8a′), 1.93–1.77 (m, 4H, H-8b, H-8′b, H-9a, H9a′), 1.76–1.55 (m, 4H, H-9b, H-9b′, H-10a, H-10a′), 1.66–1.54 (m, 2H, H-10b, H-10b′) ppm; ^13^C NMR (125 MHz, methanol-*d*_4_): *δ* = 192.0 (C=O), 164.7 (C-4′), 163.5 (C-2′), 159.3 (C-4), 144.3 (C-β), 132.9 (C-6′), 132.2 (C-2, C-6), 128.2 (C-1), 118.1 (C-α), 116.7 (C-5), 116.5 (C-3), 114.4 (C-1′), 108.5 (C-5′), 104.0 (C-3′), 96.1 (C-7′), 94.7 (C-7), 62.9 (C-11′), 62.1 (C-11), 30.6 (C-8′), 29.9 (C-8), 25.4 (C-10′), 24.9 (C-10), 19.7 (C-9′), 18.5 (C-9) ppm; HRMS (ESI-MS): *m/z* = 447.1771 ([M+Na]^+^), 425.1956 ([M+H]^+^, calcd. for C_25_H_28_O_6_ 425.1880).

### (*E*)-3-[3,4-Bis(tetrahydro-2H-pyran-2-yloxy)phenyl]-1-[2-hydroxy-4-(tetrahydro-2H-pyran-2-yloxy)phenyl]prop-2-en-1-one (**8a**, C_30_H_36_O_8_)

Compound **2a** (4.24 mmol) and 8.48 mmol of **6a** were dissolved in 10 cm^3^ of methanol at 35 °C under reflux. Subsequently, a solution of 20 cm^3^ of methanol containing barium hydroxide octahydrate (16.96 mmol) was added dropwise to the reaction mixture. The reaction mixture was stirred for 24 h and the progress was monitored by TLC. Then it was concentrated in vacuum, quenched with HCl (0.1 M), and extracted with ethyl acetate. The organic layer was separated, dried over Na_2_SO_4_, and then concentrated under vacuum. The residue yielded the crude chalcone **8a** as a yellow powder which was a mixture of stereoisomers and was used for the next step without any further purification. NMR data have not been recorded from the unpurified reaction mixture. HRMS (ESI-MS): *m/z* = 547.2291 ([M+Na]^+^), 525.2472 ([M+H]^+^).

### (*E*)-1-(2,4-Dihydroxyphenyl)-3-(4-hydroxyphenyl)-prop-2-en-1-one (**9**, ISO, C_15_H_12_O_4_)

**7a** (1.26 mmol) was dissolved in 50 cm^3^ of methanol. Subsequently pyridinium *para*-toluenesulfonate (0.062 mmol) was added and the reaction was stirred under reflux at 50 °C and the progress was monitored by TLC. Then the reaction mixture was directly concentrated under vacuum and purified by flash column chromatography (dichloromethane/methanol 9.5:0.5). ^1^H NMR (500 MHz, methanol-*d*_4_): *δ* = 7.98 (d, *J* = 9.1 Hz, 1H, H-6′), 7.80 (d, *J* = 15.4 Hz, 1H, H-β), 7.62 (d, *J* = 8.9 Hz, 2H, H-2, H-6), 7.60 (d, *J* = 15.4 Hz, 1H, H-α), 6.87 (d, *J* = 8.9 Hz, 2H, H-3, H-5), 6.42 (dd, *J* = 8.8, 2.5 Hz, 1H, H-5′), 6.29 (d, *J* = 2.5 Hz, 1H, H-3′) ppm; ^13^C NMR (125 MHz, methanol-*d*_4_): *δ* = 193.7 (C=O), 167.6 (C-4′), 166.5 (C-2′), 161.7 (C-4), 145.8 (C-β), 133.5 (C-6′), 131.9 (C-2, C-6), 128.0 (C-1), 118.5 (C-α), 117.0 (C-3, C-5), 114.8 (C-1′), 109.2 (C-5′), 103.9 (C-3′) ppm; FT-IR (KBr): $\bar{v}$ = (C–OH) 3300, (C=O) 1640, (C=C) 1600, 1587, 1520 cm^−1^; HRMS (ESI-MS): *m/z* = 279.0627 ([M+Na]^+^), 257.0807 ([M+H]^+^, calcd. for C_15_H_12_O_4_ 257.0730).

### (*E*)-1-(2,4-Dihydroxyphenyl)-3-(3,4-dihydroxyphenyl)prop-2-en-1-one (**10**, BUT, C_15_H_12_O_5_)

**8a** (1.26 mmol) was dissolved in 50 cm^3^ of methanol. Subsequently pyridinium *para*-toluenesulfonate (0.062 mmol) was added and the reaction was stirred under reflux at 50 °C and the progress was monitored by TLC. Then the reaction mixture was directly concentrated under vacuum and purified by column flash chromatography (Sephadex LH-20, dichloromethane/methanol 9.5:0.5). ^1^H NMR (500 MHz, methanol-*d*_4_): *δ* = 7.95 (d, *J* = 9.2 Hz, 1H, H-6′), 7.77 (d, *J* = 15.4 Hz, 1H, H-β), 7.53 (d, *J* = 15.4, 1H, H-α), 7.19 (d, *J* = 2.2 Hz, 1H, H-2), 7.12 (dd, *J* = 8.0, 2.2 Hz, 1H, H-6), 6.84 (d, *J* = 8.0 Hz, 1H, H-5), 6.42 (dd, *J* = 8.8, 2.5 Hz, 1H, H-5′), 6.29 (d, *J* = 2.5 Hz, 1H, H-3′) ppm; ^13^C NMR (125 MHz, methanol-*d*_4_): *δ* = 191.9 (C=O), 166.0 (C-4′), 164.4 (C-2′), 148.4 (C-5), 145.3 (C-4), 144.5 (C-β), 131.8 (C-6′), 126.9 (C-1), 122.1 (C-6), 116.8 (C-α), 115.1 (C-5), 114.2 (C-2), 113.1 (C-1′), 107.7 (C-5′), 102.4 (C-3′) ppm; FT-IR (KBr): $\bar{v}$ = (C–OH) 3300, (C=O) 1640, (C=C) 1600, 1587, 1520 cm^−1^; HRMS (ESI-MS): *m/z* = 293.1756 ([M−2H^+^+Na^+^]), 271.0617 ([M−H]^+^, calcd. for C_15_H_11_O_5_ 271.0611).

### Bis[(*E*)-1-(2,4-dihydroxyphenyl)-3-(4-hydroxyphenyl)prop-2-en-1-one]copper ([Cu(ISO)_2_], C_30_H_22_CuO_8_)

ISO (0.78 mmol) was dissolved in 15 cm^3^ of methanol under reflux. Subsequently sodium methoxide (0.8 mmol) was added to the reaction mixture. After a few minutes a solution of 15 cm^3^ methanol containing copper acetate monohydrate (0.35 mmol) was added dropwise and the color of the reaction mixture changed from orange to red-brown. The reaction mixture was stirred over night under reflux at 35 °C, then raised to room temperature and a brown precipitate was formed, which was filtered, washed with diethyl ether, and analyzed via mass spectrometry and IR spectroscopy. Yield: 52 %; FT-IR (KBr): $\bar{v}$ = (C–OH) 3300, (C=O) 1610, (C=C) 1600, 1587, 1520, (Cu–O) 500 cm^−1^; HRMS (ESI-MS): *m/z* = 573.0612 [2 ISO + ^63^Cu^2+^ + e^−^], 575.0604 ([2 ISO + ^65^Cu^2+^ + e^−^], calcd. for C_30_H_22_CuO_8_ 573.0605).

### Bis[(*E*)-1-(2,4-dihydroxyphenyl)-3-(3,4-dihydroxyphenyl)prop-2-en-1-one]copper ([Cu(BUT)_2_], C_30_H_20_CuO_10_)

BUT (0.36 mmol) was dissolved in 5 cm^3^ of methanol at reflux. Subsequently a solution containing 35 mg of copper acetate monohydrate (0.17 mmol) was added dropwise. The solution was stirred under reflux for 1 h, then a red/brown precipitate was formed, which was filtered, washed with diethyl ether, and analyzed via mass spectrometry and IR spectroscopy. Yield: 50 %; FT–IR (KBr): $\bar{v}$ = (C–OH) 3250, (C=O) 1610, (C=C) 1600, 1570, 1490, (Cu–O) 500 cm^−1^; HRMS (ESI-MS): *m/z* = 603.0362 ([2 BUT–4H + ^63^Cu^2+^], calcd. for C_30_H_20_CuO_10_ 603.034), 605.0404 [2 BUT–4H + ^65^Cu^2+^].

### Bis[(*E*)-1-(2,4-dihydroxyphenyl)-3-(4-hydroxyphenyl)prop-2-en-1-one]zinc ([Zn(ISO)_2_], C_30_H_22_ZnO_8_)

ISO (0.195 mmol) was dissolved in 5 cm^3^ of acetonitrile. After a few minutes a solution of 2.5 cm^3^ acetonitrile containing zinc chloride (0.09 mmol) was added. The reaction mixture was stirred at room temperature for 24 h. The solvent was removed under reduced pressure at room temperature to half of the initial volume. After a few hours an orange precipitate was formed, which was filtered, washed with diethyl ether, dried in air, and analyzed via mass spectrometry and IR spectroscopy. Yield: 53 %; FT–IR (KBr): $\bar{v}$ = (C–OH) 3300, (C=O) 1610, (C=C) 1600, 1587, 1520, 500 (Cu–O) cm^−1^; HRMS (ESI-MS): *m/z* = 575.0676 ([2 ISO + ^63^Zn^2+^ + H]^+^, calcd. for [C_30_H_22_ZnO_8_ + H]^+^ 575.0679), 577.0656 ([2 ISO +^65^Zn^2+^ + H])^+^.

### Bis[(*E*)-1-(2,4-dihydroxyphenyl)-3-(3,4-dihydroxyphenyl)prop-2-en-1-one]zinc ([Zn(BUT)_2_], C_30_H_22_ZnO_8_)

BUT (0.2 mmol) was dissolved in 5 cm^3^ of acetonitrile. After a few minutes a solution of 2.5 cm^3^ acetonitrile containing zinc chloride (0.09 mmol) was added. The reaction mixture was stirred at room temperature for 24 h. After few hours a yellow precipitate was formed, which was filtered, washed with diethyl ether, dried in air, and analyzed via mass spectrometry and IR spectroscopy. Yield: 50 %; FT–IR (KBr): $\bar{v}$ = (C–OH) 3300, (C=O) 1610, (C=C) 1600, 1587, 1520 (Cu–O) 500 cm^−1^; HRMS (ESI-MS): *m/z* = 607.0576 ([2 BUT + ^63^Zn^2+^ + H]^+,^ calcd. for [C_30_H_22_ZnO_8_ + H]^+^ 607.0577), 609.0564 [2 ISO + ^65^Cu^2+^ + H].

### Spectroscopic data: Job’s plot

For the Job’s plot (method of continuous variations) stock solutions of the polyhydroxychalcones **9** and **10** and Cu(OAc)_2_, Zn(OAc)_2_ were prepared in methanol-*d*_4_. For each polyhydroxychalcone/divalent cations system, eleven 5 mm high-precision NMR sample tubes were filled with 0.6 cm^3^ of the compounds in the ratios [cation]/[polyhydroxychalcone] = 0:10, 1:9 up to 9:1, and 10:0, respectively. The total concentration was kept constantly at 13 mM (600 mm^3^).

### Antioxidant activity (DPPH radical scavenging method)

The antioxidant activity test is conducted according to the method described by Ferrari et al. [48]. The test is based on the usage of 2,2-diphenyl-1-picrylhydrazyl, which is a nitrogen-centered stable radical that gives a specific absorption with a maximum at 517 nm. The free radical species DPPH reacts with the antioxidant compound and is consequently reduced to the non-radical species which produces a change in absorption [51]. The resulting decrease in absorbance was used to quantify the antioxidant activity. 1 cm^3^ of 6 × 10^−5^ mM DPPH radical solution is prepared in methanol and mixed with a variable amount (10, 25, 50, 75 mm^3^, etc.) of a methanolic solution containing the system metal-chalcone 1.2 mM. Immediately the absorbance of the mixture is measured every second up to 30 min at 517 nm. For the baseline control 1 cm^3^ of methanol was used. The percentage of inhibition of the DPPH radical was calculated for each sample referring to the following formula in accordance to Ref. [48]:

$$\% In = \frac{{A_{0} - A_{t} }}{{A_{0} }} \times 100 ,$$

where *A*_0_ is the absorbance of the control (DPPH radical) at time 0 and *A*_*t*_ is the absorbance of the mixture DPPH-antioxidant at time *t* (30 min). All determinations were performed in triplicate and the values of absorbance were corrected considering the factor of dilution.

### Solubility and kinetic stability

To test the solubility of [Cu(ISO)_2_], [Zn(ISO)_2_], [Cu(BUT)_2_], and [Zn(BUT)_2_] in water, we used the method of the shaken-flask. 100 mm^3^ stock solution of each compound in methanol (52 mM) were mixed in a vessel with regular addition of 10 mm^3^ of water and then shaken. The procedure was repeated until the formation of precipitate was observed.

The chemical stability of the four complexes was investigated at 37 °C in the darkness applying UV–Vis spectroscopy by measuring the change in absorbance in the 200–600 nm range over an overall period of 16 h. Then 50 µM solutions of the complexes were prepared in DMSO and solubilized in a 0.1 M TRIS–HCl buffer (pH 7.4). Spectra were recorded every 30 min. All profiles were linearized by a hyperbolic function (Eq. 1), which represents an empirical model that well describes drug decomposition or release [51]:

1 $$\frac{t}{{f_{\% } }} = at + b ,$$

where *f*_%_ is the fraction of residual compound at time *t* (min) expressed as a percentage referred to starting concentration at time zero.

## Electronic supplementary material

Below is the link to the electronic supplementary material.

Supplementary material 1 (DOCX 634 kb)

## References

[1] Johnson LJ, Meacham SL, Kruskall LJ (2003). J Agromed.

[2] Moon JY, Lee S, Jeong S, Kim J-C, Ahn KS, Mosaddik A, Cho SK (2013). J Korean Soc Appl Biol Chem.

[3] Feeney L, Berman ER (1976). Invest Ophthalmol.

[4] Lobo V, Patil A, Phatak A, Chandra N (2010). Pharmacogn Rev.

[5] Ávila HP, Smânia EDFA, Monache FD, Jr Smânia A (2008). Bioorg Med Chem.

[6] Cushnie TPT, Lamb AJ (2005). Int J Antimicrob Agents.

[7] Yang M, Li N, Li F, Zhu Q, Liu X, Han Q, Wang Y, Chen Y, Zeng X, Lv Y, Zhang P, Yang C, Liu Z (2013). Int Immunopharmacol.

[8] Rizvi SUF, Ahmad M, Bukhari MH, Montero C, Chatterjee P, Detorio M, Schinazi RF (2014). Med Chem Res.

[9] De Meyer N, Haemers A, Mishra L, Pandey HK, Pieters LA, Vanden Berghe AD, Vlietinck AJ (1991). J Med Chem.

[10] Gao X, Wang W, Wei S, Li W (2009). Zhongguo Zhongyao Zazhi.

[11] Kim HP, Son KH, Chang HW, Kang SS (2004). J Pharmacol Sci.

[12] Farhoosh R (2005). Food Chem.

[13] Nishida J, Kawabata J (2006). Biosci Biotechnol Biochem.

[14] Bentes LA, Borges RS, Monteiro WR, De Macedo LGM, Alves CN (2011). Molecules.

[15] Kim BT, O KJ, Chun JC, Hwang KJ (2008) Bull Korean Chem Soc 29:1125

[16] Grazul M, Budzisz E (2009). Coord Chem Rev.

[17] de Souza RFV, Sussuchi EM, De Giovan WF (2003). Synth React Inorg Met Chem.

[18] Pereira RMS, Andrades NED, Paulino N, Sawaya ACHF, Eberlin MN, Marcucci MC, Favero GM, Novak EM, Bydlowski SP (2007). Molecules.

[19] Panhwar QK, Memon S (2014). Chem Pap.

[20] Devi JM, Tharmaraj P, Ramakrishnan SK, Ramachandran K (2008). Mater Lett.

[21] de Souza RFV, de Giovani WF (2004). Redox Rep.

[22] Cornard JP, Merlin JC (2003). J Mol Struct.

[23] Palaniandavar M, Natarajan C (1980). Aust J Chem.

[24] Barry NPE, Sadler PJ (2013). Chem Commun.

[25] Kupcewicz B, Sobiesiak K, Malinowska K, Koprowska K, Czyz M, Keppler B, Budzisz E (2013). Med Chem Res.

[26] Bratu MM, Birghila S, Miresan H, Negreanu-Pirjol T, Prajitura C, Calinescu M (2014). Rev Chim.

[27] Qian J-Z, Wang B-C, Fan Y, Tan J, Huang HJ (2014). J Coord Chem.

[28] Chrichton RR (2012). Biological inorganic chemistry. A new introduction to molecular structure and function.

[29] Sumathi S, Tharmaraj P, Sheela CD, Ebenezer R (2011). J Coord Chem.

[30] Severi F, Benvenuti S, Costantino L, Vampa G, Melegafi M, Antolini L (1998). Eur J Med Chem.

[31] Van Duuren BL (1969). Ann N Y Acad Sci.

[32] Grealis JP, Müller-Bunz H, Ortin Y, Casey M, McGlinchey MJ (2013). Eur J Org Chem.

[33] Ninomiya M, Tanaka K, Tsuchida Y, Muto Y, Koketsu M, Watanabe K (2011). J Med Chem.

[34] González-Calderón D, González-González CA, Fuentes-Benítez A, Cuevas-Yáñez E, Corona-Becerril D, González-Romero C (2013). Tetrahedron Lett.

[35] Lee S, Kim JK, Lee JG, Lee JS, Leem M, Chung YH, Song YO, Suh H (2001). Bull Korean Chem Soc.

[36] Job P (1928). Ann Chim.

[37] Kupcewicz B, Grazul M, Lorenz I-P, Mayer P, Budzisz E (2011). Polyhedron.

[38] Jungbluth G, Rühling I, Ternes W (2000). J Chem Soc Perkin Trans.

[39] Le Nest G, Caille O, Woudstra M, Roche S, Burlat B, Belle V, Guigliarelli B, Lexa D (2004). Inorg Chim Acta.

[40] Roshal AD, Munos O, Sakhno TV, Buadon MT (2002). Chem Heterocycl Compd.

[41] McWhinnie WR (1965). J Inorg Nucl Chem.

[42] Woźnicka E, Kopacz M, Umbreit M, Kłos J (2007). J Inorg Biochem.

[43] Panhwar QK, Memon S, Bhanger MI (2010). J Mol Struct.

[44] Cornard JP, Merlin JC (2002). J Inorg Biochem.

[45] Pirc ET, Modec B, Cer-Kerčmar K, Bukovec P (2014). Monatsh Chem.

[46] Bandgar BP, Gawande SS, Bodade RG, Gawande NM, Khobragade CN (2009). Bioorg Med Chem.

[47] Mira L, Fernandez MT, Santos M, Rocha R, Florencio MH, Jennings KR (2002). Free Radic Res.

[48] Ferrari E, Asti M, Benassi R, Pignedoli F, Saladini M (2013). Dalton Trans.

[49] Pekal AM, Biesaga K, Pyrzynska K (2011). Biometals.

[50] Ferrari E, Pignedoli F, Imbriano C, Marverti G, Basile V, Venturi E, Saladini M (2011). J Med Chem.

[51] Arcos D, López-Noriega A, Ruiz-Hernández E, Terasaki O, Vallet-Regí M (2009). Chem Mater.

