# Supplementary material for: Synthesis, characterization, and antioxidant activity of Zn2+ and Cu2+ coordinated polyhydroxychalcone complexes
Source: Monatsh Chem. 2016 Aug 29;147(11):1871–81. doi: 10.1007/s00706-016-1822-7 (PMC5063914; doi:10.1007/s00706-016-1822-7)
Supplement: Supplementary file 1 — Supplementary material 1 (DOCX 634 kb) [file 706_2016_1822_MOESM1_ESM.docx]

Supporting Information

Synthesis, characterization and antioxidant activity of Zn^2+^ and Cu^2+^ coordinated polyhydroxychalcone complexes

**Chiara Sulpizio^1^, Simon T.R. Müller^1^, Qi Zhang^1^, Lothar Brecker^2^, Annette Rompel^1^**

^1^ [Institut für Biophysikalische Chemie](http://online.univie.ac.at/inst?inum=A525), [Fakultät für Chemie](http://chemie.univie.ac.at/aktuelles/), Universität Wien, Althanstraße 14, 1090 Wien, Austria

^2^ [Institut für Organische Chemie](http://online.univie.ac.at/inst?inum=A521), [Fakultät für Chemie](http://chemie.univie.ac.at/aktuelles/), Universität Wien, Währinger Straße 38, 1090 Wien, Austria

**Kinetic Stability**

**
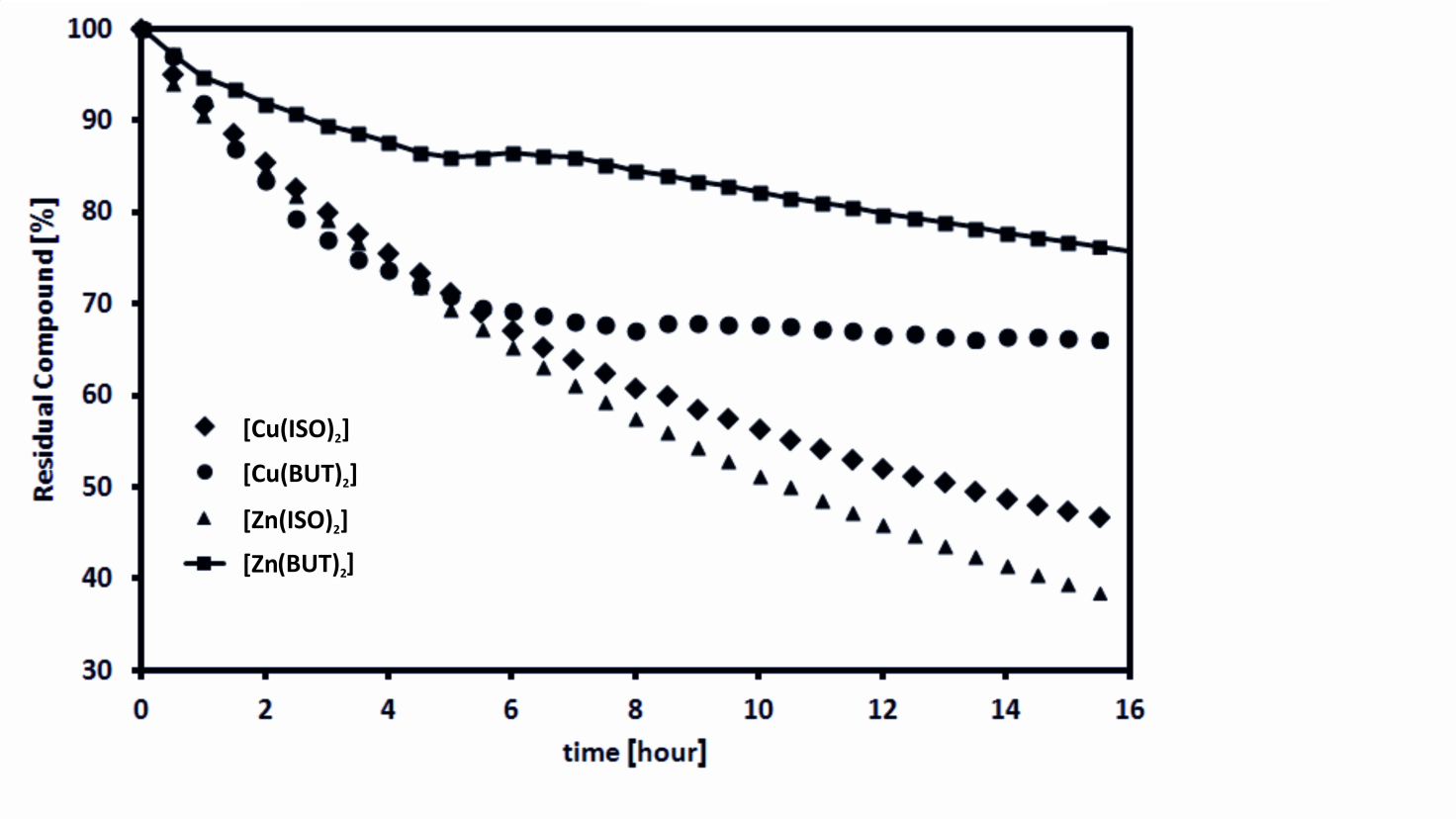
**

**figure S1.** Decomposition kinetics profiles of **[Cu(ISO)_2_]**, **[Cu(BUT)_2_],** **[Zn(ISO)_2_]**, **[Zn(BUT)_2_]** compounds in buffered aqueous solution (TRIS-HCl pH = 7.4) at 37 °C in darkness over a period of 16 h. Residual concentration is expressed as percentage with respect to concentration at *time zero.* Concentrations were determined

by reading absorbance at 385 nm.

**
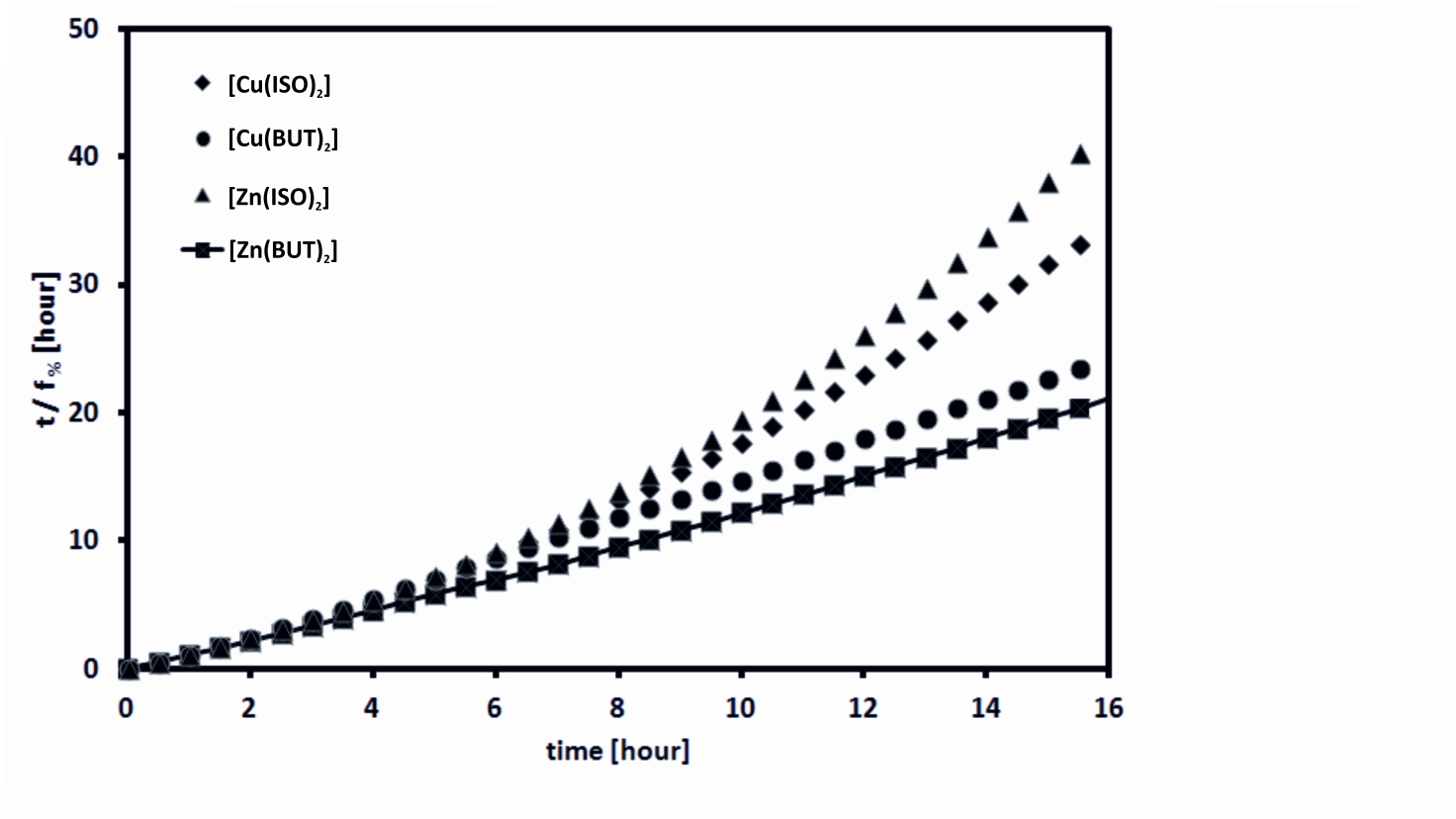
figureS2.** Linearization of kinetics profiles of **[Cu(ISO)_2_]**, **[Cu(BUT)_2_],** **[Zn(ISO)_2_]**, **[Zn(BUT)_2_]** compounds by the hyperbolic function *t*/*f*% = *at* + *b*, where *t* is time (min) and *f*% represents concentration as residual percentage

TableS1: Linear fittings for kinetics Profiles obtained using the function : *t/f= at+b^a^*

| Compd. | *a* | (1/*a*) | *b* | (-1/*b*) | *R*^2^ |
| --- | --- | --- | --- | --- | --- |
| [Cu(ISO)_2_] | 1,6431 | 0,60 | -0,7906 | 1,25 | 0,9891 |
| [Cu(BUT)_2_] | 1,546 | 0,64 | -0,5784 | 1,72 | 0,9996 |
| [Zn(ISO)_2_] | 1,7255 | 0,57 | -0,9276 | 1,07 | 0,985 |
| [Zn(BUT)_2_] | 1,3427 | 0,74 | -0,8036 | 1,24 | 0,9966 |

*^a^*Equation parameters (*a* and *b*) and statistical parameters *R^2^* were extracted by a least-square fitting on the experimental UV-Vis results.”
